# Effects of auricular stimulation on weight- and obesity-related parameters: a systematic review and meta-analysis of randomized controlled clinical trials

**DOI:** 10.3389/fnins.2024.1393826

**Published:** 2024-08-06

**Authors:** Kevin Hua, Taras Usichenko, Mike Cummings, Miriam Bernatik, Stefan N. Willich, Benno Brinkhaus, Joanna Dietzel

**Affiliations:** ^1^Institute for Social Medicine, Epidemiology and Health Economics, Charité - University Medicine, Corporate Member of Freie Universität Berlin, Humboldt-Universität zu Berlin, and Berlin Institute of Health, Berlin, Germany; ^2^Department for Anaesthesiology, University Hospital Greifswald, Greifswald, Germany; ^3^Department of Anesthesia, McMaster University, Hamilton, ON, Canada; ^4^British Medical Acupuncture Society, London, United Kingdom; ^5^International Society of Chinese Medicine, Munich, Germany

**Keywords:** transauricular vagus nerve stimulation, ear-acupuncture, obesity, blood lipids, systematic review, meta-analysis

## Abstract

**Background:**

Over the last three decades, the number of randomized controlled trials (RCTs) using stimulation of auricular vagal sensory nerves by means of electrical stimulation, auricular acupuncture, or acupressure to support weight loss has increased markedly. This systematic review focuses on the effects of auricular stimulation (AS) on anthropometric parameters and obesity-related blood chemistry.

**Methods and analysis:**

The following databases were searched until November 2021: MEDLINE (PubMed), EMBASE, Cochrane Central Register of Controlled Trials (CENTRAL), ISI Web of Science, and Scopus Database. Data collection and analysis were conducted by two reviewers independently. Quality and risk assessment of included studies was performed using the risk of bias tool of the Cochrane Handbook, and the meta-analysis of the effect of the most frequently assessed biomarkers was conducted using the statistical software RevMan.

**Results:**

The full texts of 1,274 studies were screened; 22 contained data on obesity-related outcomes, and 15 trials with 1,333 patients were included in the meta-analysis. The overall quality of the included trials was moderate. AS significantly reduced body mass index (BMI) (mean difference (MD) = −0.38 BMI points, 95% CI (−0.55 to −0.22), *p* < 0.0001), weight (MD = −0.66 kg, 95% CI (−1.12 to −0.20), *p* = 0.005), waist circumference (MD = −1.44 cm, 95% CI (−2.69 to −0.20), *p* = 0.02), leptin, insulin, and HOMA insulin resistance compared to controls. No significant reduction was found in body fat, hip circumference, ratio of waist/hip circumference, cholesterol, LDL, triglycerides, adiponectin, ghrelin, and glucose levels. The AS was safe throughout the trials, with only minor adverse reactions.

**Conclusion:**

The study results suggest that a reduction of weight and BMI can be achieved by AS in obese patients; however, the size of the effect does not appear to be of clinical relevance. The effects might be underestimated due to active sham trials.

**Systematic review registration:**

https://www.crd.york.ac.uk/prospero/display_record.php?ID=CRD42021231885.

## 1 Introduction

### 1.1 Background

Obesity is a major public health concern worldwide, with a growing number of individuals affected. Despite the availability of various weight loss interventions, many individuals struggle to achieve and maintain a healthy weight. One treatment for obesity is AS, which has been shown in clinical trials to be effective in reducing stress and anxiety (Usichenko et al., 2022). Embedded ear needles were popularized in the ‘70s and ‘80s for weight loss (Schatz, 1975), which might have propagated the conduct of many clinical trials using auricular therapy for various conditions (insomnia, cocaine addiction, back pain, and epilepsy; Gates et al., 2006; Lan et al., 2015; Liu et al., 2018; Moura et al., 2019; Mendonça et al., 2020; Yap et al., 2020). Currently, it is presumed that AS exerts its effects through the involvement of cranial nerves V, VII, and X, which lead to the modulation of brain areas involved in stress response, such as the limbic system, locus coeruleus, and hypothalamus (Qu et al., 2014; Frangos et al., 2015). The main response is seen in connection to the vagal nerve, which presents on the auricle (Arnold’s nerve) and can be directly stimulated in the cymba and the region around the meatus acusticus externus (Peuker and Filler, 2002; Peuker, 2003). This study aimed to provide a comprehensive and systematic review of RCTs assessing the effects of AS on weight reduction and obesity-related parameters such as waist circumference, waist-to-hip ratio, and blood lipids in obese patients. The study aimed to provide a better understanding of the potential benefits and limitations of AS as a treatment strategy for weight loss in obesity and its effect on obesity-related parameters.

### 1.2 Objectives

Systematic review and meta-analysis to evaluate the effects of AS on obesity-related parameters and the safety of AS.

## 2 Methods

The systematic review protocol has been registered on PROSPERO ID CRD42020184795. The systematic review and meta-analysis were conducted in accordance with the Cochrane Handbook for Systematic Reviews of Interventions and the PRISMA guidelines. The protocol aimed at an investigation of the effects of AS on a large array of biomarkers and other objective outcomes. This study focuses on obesity-related outcomes.

### 2.1 Eligibility criteria for included trials in the review

#### 2.1.1 Types of trials

Only RCTs with a full text published in European languages were eligible for the review.

#### 2.1.2 Types of participants

Obese patients have no restrictions on age, sex, ethnicity, or further health conditions. Obesity was defined as a BMI greater than 25 (Engin, 2017). If other ethnic groups are investigated, adjusted BMI will be applied. For Asian Americans, the adjusted BMI for obesity is defined as a BMI greater than 23 (Jih et al., 2014).

#### 2.1.3 Types of interventions

We included all RCTs in which AS was used alone or in addition to further weight loss measures. All interventions were eligible, including traditional AS (i.e., auricular acupuncture, auricular acupuncture with electric stimulation, and auricular acupressure) as well as related techniques such as transcutaneous electrical stimulation of the auricular nerve (tVNS) [synonym: transauricular vagus nerve stimulation (taVNS)] or cranial electrotherapeutic stimulation (CES, on the ear lobule). All control conditions (sham or placebo stimulation, diets, exercise, routine care, etc.) were included. We excluded studies that compared one type of AS technique only with another AS technique.

#### 2.1.4 Types of outcome measures

The main outcomes were body weight and BMI, body fat, waist circumference, hip circumference, waist/hip circumference ratio, blood lipids, cholesterol, and obesity-related blood chemistry (HbA1c, blood glucose, leptin, ghrelin, and homeostasis model assessment index). All biomarkers, that were reported with results were extracted and evaluated. The continuous data were pooled in a meta-analysis, while the non-continuous data were evaluated descriptively. Adverse event reporting was used to analyze safety.

### 2.2 Search methods for identification of trials

#### 2.2.1 Electronic searches

Two researchers (JD and KH) searched the following databases from inception until 17 November 2021: MEDLINE (PubMed), EMBASE, Cochrane Central Register of Controlled Trials (CENTRAL), ISI Web of Science, and Scopus Database. The complete search strategy is listed in Table 1.

**Table 1:** Search strategy for the MEDLINE database.

#Number	Search term (title/abstract) (combined with OR)
1	Randomized controlled trial
2	Controlled clinical trial
3	Randomized
4	Trial
5	RCT
AND	
6	Auricular acupuncture
7	Auricular acupressure
8	Auricular electro-acupuncture
9	Auricular stimulation
10	Auriculotherapy
11	Ear acupuncture
12	taVNS
13	Auricular vagus nerve stimulation
14	tVNS
15	Transcutaneous vagus nerve stimulation
16	Transauricular vagus nerve stimulation
17	Percutaneous auricular vagus nerve stimulation
18	Auricular laser stimulation
19	CES
20	Cranial electrotherapy stimulation

### 2.3 Data extraction and management

#### 2.3.1 Trial identification

**Figure 1:**
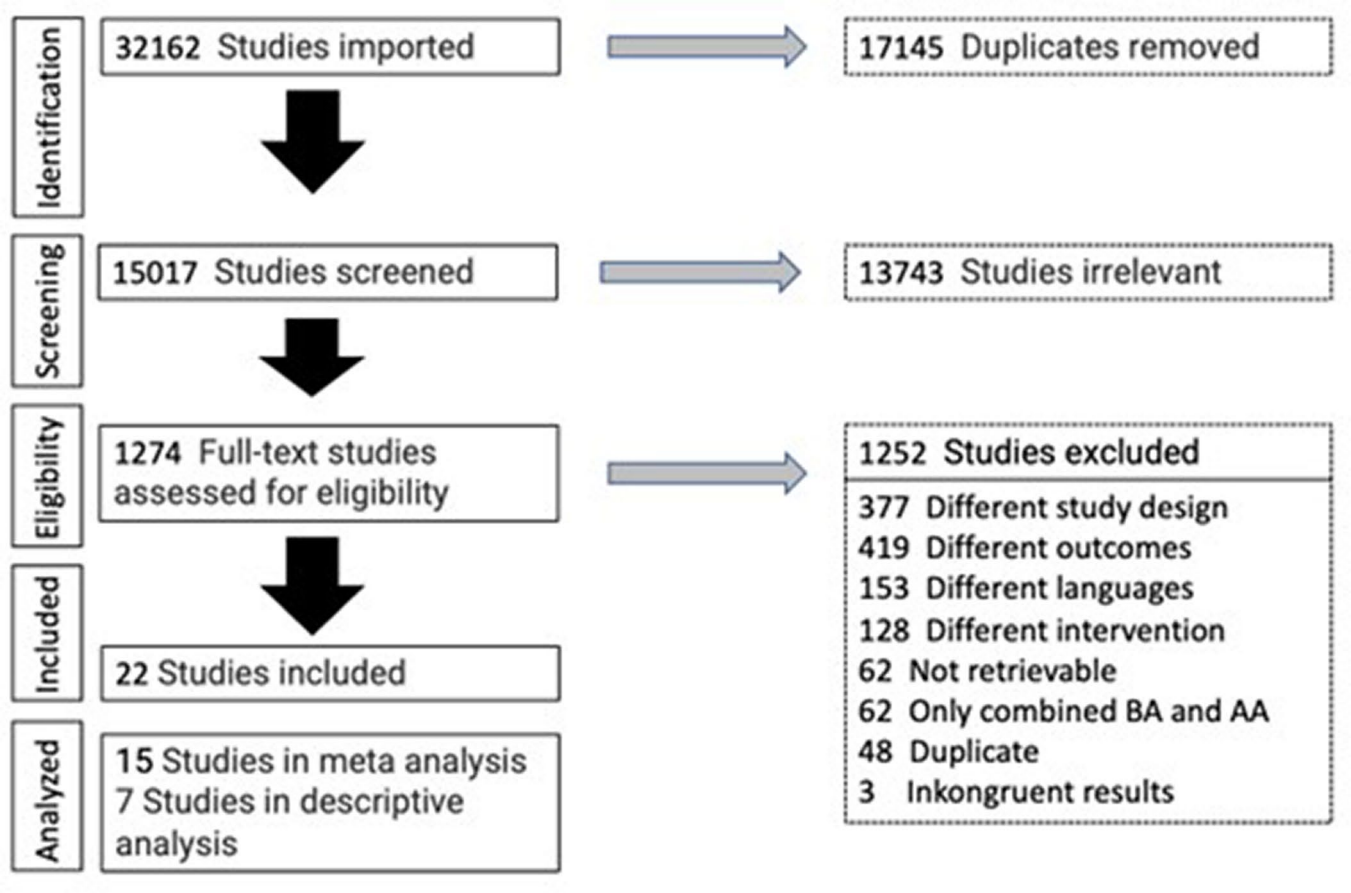
PRISMA flowchart.

Two researchers (JD and KH) independently reviewed: titles, abstracts, and full texts for suitability. Discrepancies were resolved by discussion with a third author (TU). If an article did not contain enough information to determine eligibility, we contacted the trial authors via email. The selection process is shown in the PRISMA flowchart in Figure 1.

#### 2.3.2 Data extraction and assessment of risk of bias in included trials

JD and KH independently extracted data. The methodological quality of each RCT was assessed using the risk of bias tool I recommended in the Cochrane Handbook. A consensus process was conducted before entering the data into the Review Manager software (RevMan 5.4. 2020).

#### 2.3.3 Measures of treatment effects and dealing with missing data

The results of the treatment were analyzed. In cases of multiple-arm trials, only the arms that fulfilled the inclusion criteria were chosen. The outcomes presented as continuous data were analyzed as MDs with 95% confidence intervals (CIs) or standardized mean differences (SMDs). When final means were not reported, or baseline values differed in a relevant way, changes from baseline were used in the meta-analysis. According to Cochrane Handbook Chapter 10.5.2., MD was applied. If a relevant number of data were missing, this was indicated in the risk of bias section. We did not use imputation or other strategies for missing data. For non-continuous data that were not suitable for meta-analysis, the selected effect measures were analyzed descriptively.

#### 2.3.4 Assessment of heterogeneity

Because of the broad inclusion criteria, high heterogeneity was expected. We used random-effects meta-analysis instead of fixed-effects meta-analysis when we considered high heterogeneity to be relevant. Heterogeneity was considered substantial if T^2^ was greater than zero and either I^2^ was greater than 50% or if the X^2^ test for heterogeneity yielded a low *p*-value (less than 0.10).

#### 2.3.5 Assessment of reporting biases

A funnel plot with asymmetry was examined for each of the included trials.

### 2.4 Data synthesis

Fixed-effect meta-analyses were performed initially, in cases of high heterogeneity, random-effect analyses were performed. Data not suitable for meta-analysis were reported separately. Furthermore, for each anthropometric parameter, a GRADE assessment was undertaken.

#### 2.4.1 Subgroup analysis and sensitivity analysis

Subgroup analysis was performed to assess the effects of the different AS methods on the single obesity-related outcomes. Sensitivity analysis was considered for the outliers of the meta-analysis.

## 3 Results

### 3.1 Literature search and analysis

Out of 1,274 trials that were analyzed with full-text analysis, a total of 22 met the inclusion criteria (see Figure 1).

### 3.2 Data extraction and analysis

The number of trial participants, gender, age, type of intervention, and assessment method for anthropometric parameters are summarized in Table 2.

**Table 2:** Overview of eligible RCTs.

	Country	Population	Auricular intervention (*n*)	Controls (*n*)	Days of stim	Side of treatment	Auricular innervation
*Allison et al. (1995) and Peuker (2003)	United States	m/f	Apres (35)	Wrist acupressure (34)	84	Unilateral	ABVN/GAN
Hsieh (2010) and Engin (2017)	Taiwan	m/f	Apres + wr-education (27)	Sham apres + wr- education (28)/ wr- education alone (29)	53	Alternating	NR
Hsieh (2007) and Jih et al. (2014)	Taiwan	m/f	Apres + wr- education (27)	Sham apres + wr- education (28)	56	NR	ABVN/GAN/ATN
*He et al. (2012) and Cha and Park (2020)	China	f	Apres + exercise (30)	Exercise (30)	28	Alternating	ABVN/GAN/ATN
*Yeh and Yeh (2008) and Darbandi et al. (2012)	Iran	m/f	Apres + diet (43)	Sham apres + diet (34)	42	Bilateral	ABVN/GAN/ATN
*Hsieh (2010) and Yeo et al. (2014)	South Korea	m/f	Apunc + diet (22)	Sham apunc +diet (21)/ diet only (15)	56	Alternating	ABVN/GAN
*Hsieh (2007) and Yeh et al. (2015)	Taiwan	m/f	Electr. Apres + diet (36)	Sham apres + diet (34)	70	NR	ABVN/GAN/ATN
Ching et al. (2012) and Schukro et al. (2014)	Austria	f	Electr. Apunc +diet (28)	Sham electro-apunc + diet (28)	42	NR	ABVN/GAN/ATN
*Costa-Cavalcanti et al. (2018) and Cha and Park (2020)	South Korea	Children m/f	Apres +diet (31)	Sham apres +diet (34)	56	Alternating	ABVN/GAN/ATN
*Allison et al. (1995) and Ching et al. (2012)	Taiwan	m/f in-patients with schizophrenia	Apres + diet (33)	Sham + diet (39)	56	Alternating	ABVN/GAN/ATN
*He et al. (2012) and Darbandi et al. (2014)	Iran	m	Apres +diet (20)	Sham apres +diet (20)/ body electro- acup +diet (20) / sham body-apunc +diet (20)	42	Bilateral	ABVN/GAN/ATN
*Darbandi et al. (2012) and Kim et al. (2014)	South Korea	f	Apres (25)	No intervention (24)	28	Alternating	ABVN/GAN
Hsieh et al. (2011) and Yeh et al. (2015)	Taiwan	m/f	Apres+ wr- education (27)	Sham- apres + wr- education (28)	56	NR	ABVN/GAN
*Yeh and Yeh (2008) and Schukro et al. (2014)	Taiwan	m/f	Apres (19)	No intervention (19)	63	Alternating	ABVN/GAN
Qunli and Zhicheng (2005) and Kim et al. (2014)	China	m/f	Apunc (55)	body-apunc (64)/body-apunc + a. apunc (76)	28	Alternating	ABVN/GAN/ATN
*Mok et al. (1976) and Hsieh et al. (2011)	United States	m/f	Apunc (24)	sham-apunc (24)/no intervention (24)	21	Bilateral	ABVN/GAN
*Shen et al. (2009) and Lien et al. (2012)	Taiwan	f	Apunc (24)	Sham-apunc (24)/apres (24)	28	Alternating	ABVN/GAN/ATN
Shen et al. (2009) and Cayir et al. (2017)	Taiwan	f	Apunc (13)	Sham–apunc (13)	28	Alternating	ABVN/GAN/ATN
*Hsu et al. (2009) and Cayir et al. (2017)	Turkey	f	Apunc (17)	Body-apunc (21)	87	Bilateral	ABVN/GAN
*Hsu et al. (2009) and Lillingston et al. (2019)	Taiwan	f	Apunc (23)	Sham-apunc (22)	42	Alternating	ABVN/GAN/ATN
Lillingston et al. (2019) and Yeo et al. (2014)	Caribbean	f	Apunc (30)	Sham-apunc (28)	49	NR	ABVN/GAN
*Qunli and Zhicheng (2005) and Costa-Cavalcanti et al. (2018)	Brazil	m/f with osteoarthritis of knee	Apres (11)	Sham (12)	35	Bilateral	ABVN/GAN

Trials included in the meta-analysis are marked with an *. m, male; f, female; apres: acupressure; apunc: acupuncture; wr- education: weight reduction education including advice on diet, exercise, and further lifestyle modifications. NR, not reported; ABVN, auricular branch of vagus nerve; GAN, great auricular nerve; ATN, auriculo-temporal nerve.

### 3.3 Baseline characteristics

A total of 1,333 individuals were included in this systematic review. A total of 91% were female subjects. One trial did not provide any information on sex concerning the included school children (Cha and Park, 2020). The age of the individuals ranged between 10 and 62 years, with a median of 35 years. One trial examined a mixed population of obese and non-obese patients (Yeh and Yeh, 2008). Obesity was defined as a BMI >25. Two studies adjusted the cutoff for obesity because of the Asian population (Hsieh, 2007, 2010). In the subgroup analysis, we pooled only the data of the obese trial participants. One trial was conducted on psychiatric in-patients (Ching et al., 2012), and another on a sample with knee osteoarthritis (Costa-Cavalcanti et al., 2018).

### 3.4 Comparison of trial designs

Several study designs could be identified. Most of the studies were designed as two-armed studies (*n* = 16) (Allison et al., 1995; Hsieh, 2007; Yeh and Yeh, 2008; Hsu et al., 2009; Shen et al., 2009; Hsieh et al., 2011; Ching et al., 2012; Darbandi et al., 2012; He et al., 2012; Kim et al., 2014; Schukro et al., 2014; Yeh et al., 2015; Cayir et al., 2017; Costa-Cavalcanti et al., 2018; Lillingston et al., 2019; Cha and Park, 2020), followed by three-armed studies (*n* = 5) (Mok et al., 1976; Qunli and Zhicheng, 2005; Hsieh, 2010; Lien et al., 2012; Yeo et al., 2014) and four-armed studies (*n* = 1) (Darbandi et al., 2014). All the included trials were conducted on obese individuals.

### 3.5 Intervention

Thirteen RCTs used acupressure with small beads or plant seeds for stimulation (Allison et al., 1995; Hsieh, 2007, 2010; Yeh and Yeh, 2008; Hsieh et al., 2011; Ching et al., 2012; Darbandi et al., 2012, 2014; He et al., 2012; Kim et al., 2014; Yeh et al., 2015; Costa-Cavalcanti et al., 2018; Cha and Park, 2020). Auricular acupuncture with conventional needles or semipermanent needles was identified in eight studies (Mok et al., 1976; Qunli and Zhicheng, 2005; Hsu et al., 2009; Shen et al., 2009; Lien et al., 2012; Schukro et al., 2014; Yeo et al., 2014; Cayir et al., 2017; Lillingston et al., 2019). Additional electrical stimulation of the ear was used in one trial (Schukro et al., 2014). In 21 of all 22 trials, stimulation was applied in auricular regions with vagal innervation (mainly concha). One study did not provide any information about the location of AS (Hsieh, 2010).

Eleven trials used add-on AS to the use of additional weight loss interventions, such as exercise, diet, or weight reduction education, which included diet and physical exercise counseling for all treatment arms (Hsieh, 2007, 2010; Hsieh et al., 2011; Darbandi et al., 2012; Schukro et al., 2014; Yeo et al., 2014; Yeh et al., 2015). The mean duration of AS was 48 days, ranging between 21 days (Mok et al., 1976) and 87 days (Cayir et al., 2017).

### 3.6 Controls

Different control groups were identified in the included studies. The most common control group was a sham procedure (Mok et al., 1976; Hsieh, 2007, 2010; Hsu et al., 2009; Shen et al., 2009; Hsieh et al., 2011; Ching et al., 2012; Darbandi et al., 2012, 2014; Lien et al., 2012; Schukro et al., 2014; Yeo et al., 2014; Yeh et al., 2015; Costa-Cavalcanti et al., 2018; Lillingston et al., 2019; Cha and Park, 2020). Some studies have also compared AS vs. active controls (Mok et al., 1976; Hsieh, 2007, 2010; He et al., 2012; Lien et al., 2012; Wang et al., 2014; Yeo et al., 2014). These included mostly dietary instructions (Hsieh, 2007, 2010; Yeo et al., 2014) and physical exercise (He et al., 2012). Four studies also compared AS to classical body acupuncture and acupressure (Allison et al., 1995; Qunli and Zhicheng, 2005; Darbandi et al., 2014; Cayir et al., 2017). Four trials had no-intervention control (Yeh and Yeh, 2008; Kim et al., 2014).

### 3.7 Outcomes

**Figure 2:**
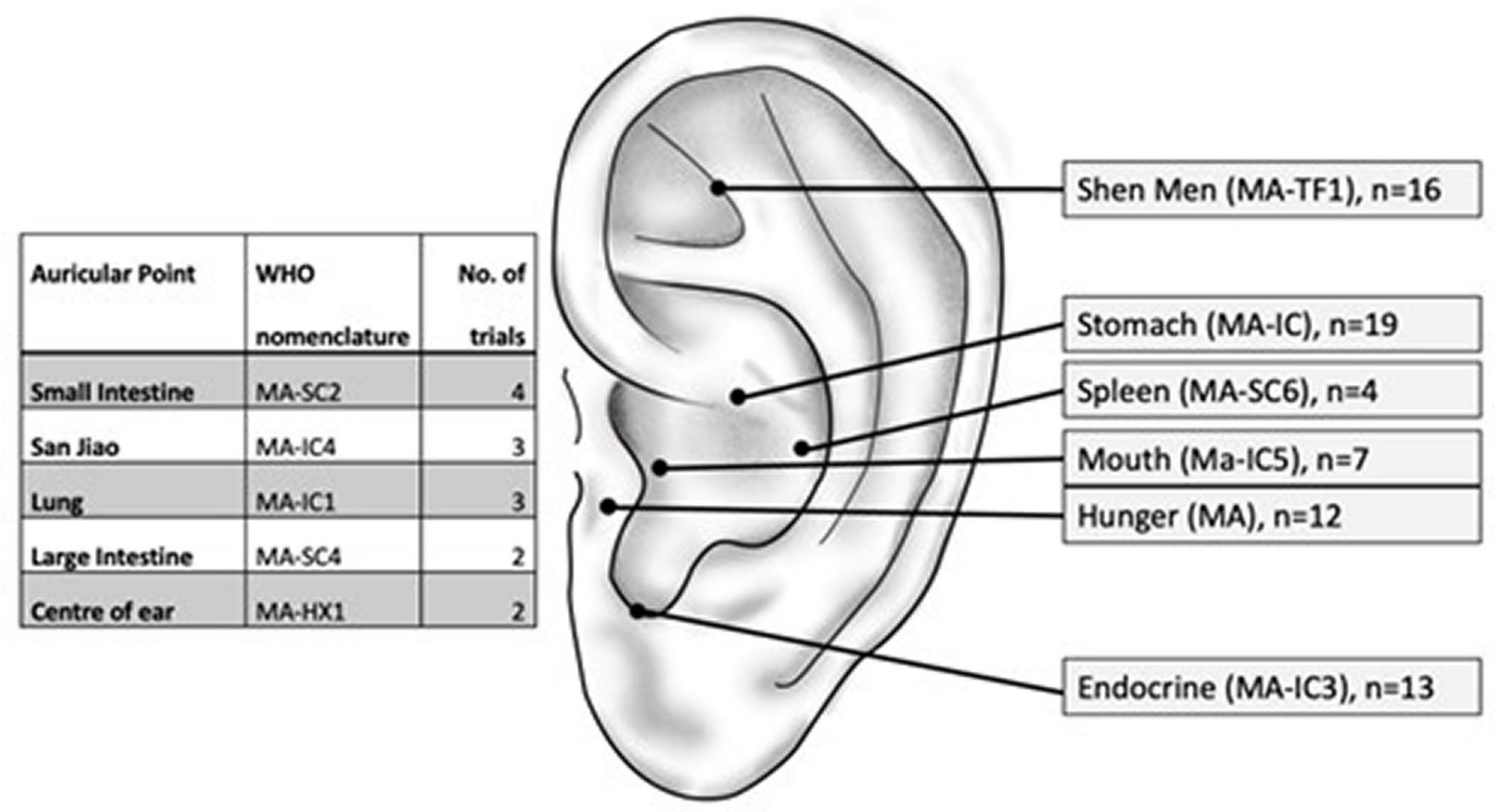
Auricular points used in obesity and blood lipid reduction trials. Not shown: points used in a single trial.

All trials applied AS on regions of vagal innervation via the auricular branch of the vagus nerve (ABVN) (see Table 1 and for most frequently used points, see Figure 2). Most trials have examined the effects of AS on anthropometric parameters. Weight (*n* = 17) (Mok et al., 1976; Allison et al., 1995; Qunli and Zhicheng, 2005; Yeh and Yeh, 2008; Hsu et al., 2009; Shen et al., 2009; Hsieh et al., 2011; Ching et al., 2012; Darbandi et al., 2012; He et al., 2012; Lien et al., 2012; Kim et al., 2014; Schukro et al., 2014; Yeo et al., 2014; Cayir et al., 2017; Lillingston et al., 2019; Cha and Park, 2020), BMI (*n* = 12) (Hsieh, 2007, 2010; Yeh and Yeh, 2008; Hsu et al., 2009; Darbandi et al., 2012, 2014; He et al., 2012; Lien et al., 2012; Kim et al., 2014; Schukro et al., 2014; Yeo et al., 2014; Cayir et al., 2017; Lillingston et al., 2019), body fat (*n* = 6) (Allison et al., 1995; Yeh and Yeh, 2008; Darbandi et al., 2012; Kim et al., 2014; Yeo et al., 2014; Cayir et al., 2017), waist circumference (*n* = 6) (Yeh and Yeh, 2008; He et al., 2012; Lien et al., 2012; Darbandi et al., 2014; Yeo et al., 2014; Cayir et al., 2017), hip circumference (*n* = 4) (Yeh and Yeh, 2008; Lien et al., 2012; Darbandi et al., 2014; Cayir et al., 2017), and the ratio of waist and hip circumference (*n* = 2) (Yeh and Yeh, 2008; Kim et al., 2014). Three trials examined the effect on glucose metabolism (Hsu et al., 2009; Lien et al., 2012; Costa-Cavalcanti et al., 2018), and a total of five studies have investigated the effects related to lipid metabolism and digestive hormones (Hsu et al., 2009; Darbandi et al., 2012; Lien et al., 2012; Yeh et al., 2015; Costa-Cavalcanti et al., 2018). Of the 22 trials included in this systematic review, 15 provided sufficient data and were eligible for meta-analysis, while 7 trials were evaluated descriptively. Forest plots are only presented for pooled outcomes of five trials and more. Forest plots of four or fewer trials can be found in the Supplementary material.

### 3.8 Body mass index

**Figure 3:**
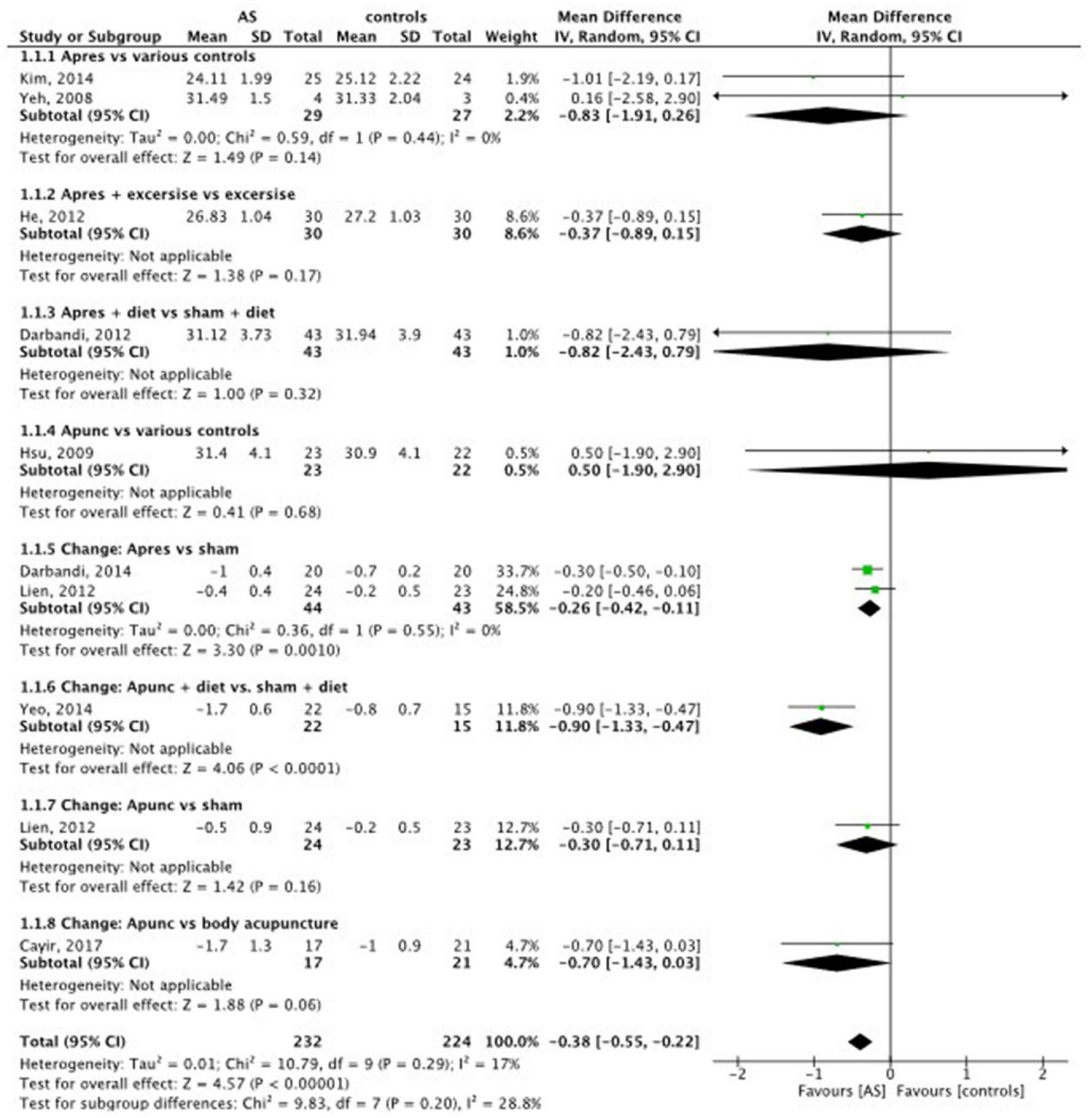
Body mass index (BMI): AS vs. controls.

A total of 10 trials with 487 individuals provided data on BMI. Compared to the control methods, AS significantly reduced BMI (MD = −0.38 BMI points, 95% CI (−0.55 to −0.22), *p* < 0.0001) (see Figure 3). The strongest BMI reduction of a mean of 1.01 points was achieved in a two-armed trial by Kim et al. (2014). South Korean female obese college students were allocated to ear acupressure or waiting lists. After 1 month of continuous and self-applied ear acupressure with diet or exercise, the acupressure group lost 3.1 kg (SD 0.73) vs. 0.2 kg (SD 1.05) (difference *p* < 0.001) and 1.23 (SD 0.34) BMI points vs. 0.15 (SD 0.45) BMI points (difference *p* < 0.001) compared to the control group in the pre-post calculation. The authors explained that satisfying results were connected to self-treatment and an increased feeling of self-efficacy. A similar reduction of weight and BMI was achieved in the sham-controlled trial of Lien et al. Taiwanese obese women received auricular true or sham acupuncture. Regular diets were maintained throughout the study. After 1 month of ear acupuncture with semipermanent needles, the real acupuncture group had a mean of 1.3 kg (SD 2.2) vs. 0.6 kg (SD 1.4) and a BMI of 0.5 points (SD 0.9) vs. 0.2 points (SD 0.5) in the sham acupuncture group.

### 3.9 Weight

**Figure 4:**
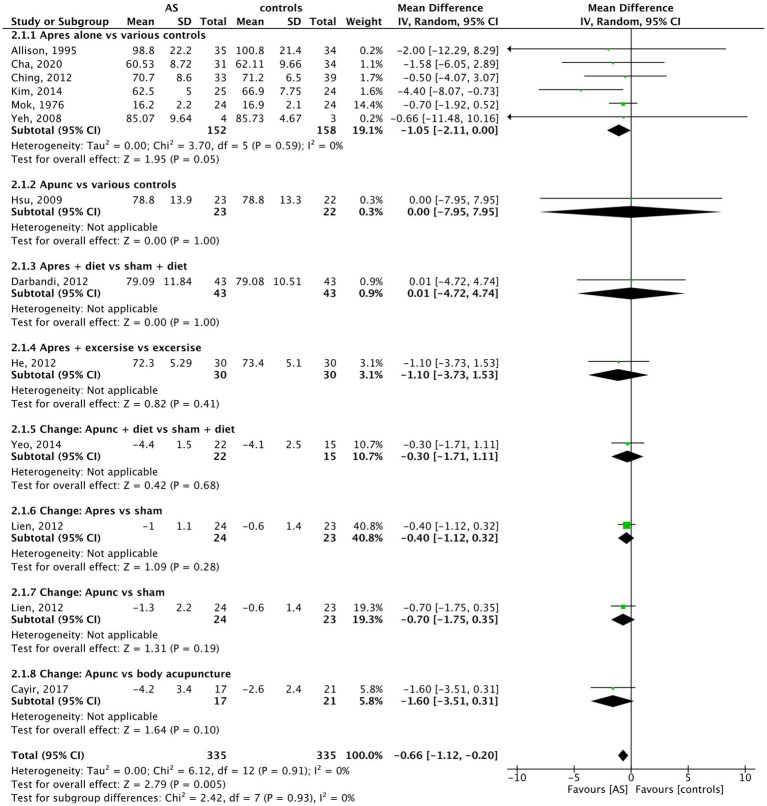
Weight: AS vs. controls.

In 12 trials with data on the body weight of 655 individuals, compared to the control methods, AS led to a significant reduction of body weight (MD = −0.66 kg, 95% CI (−1.12 to −0.20), *p* = 0.005) (see Figure 4).

No significant difference, despite a low-calorie diet, could be achieved with add-on acupressure compared to add-on sham acupressure in Iranian obese men after 6 weeks of treatment (Darbandi et al., 2014).

### 3.10 Body fat

**Figure 5:**
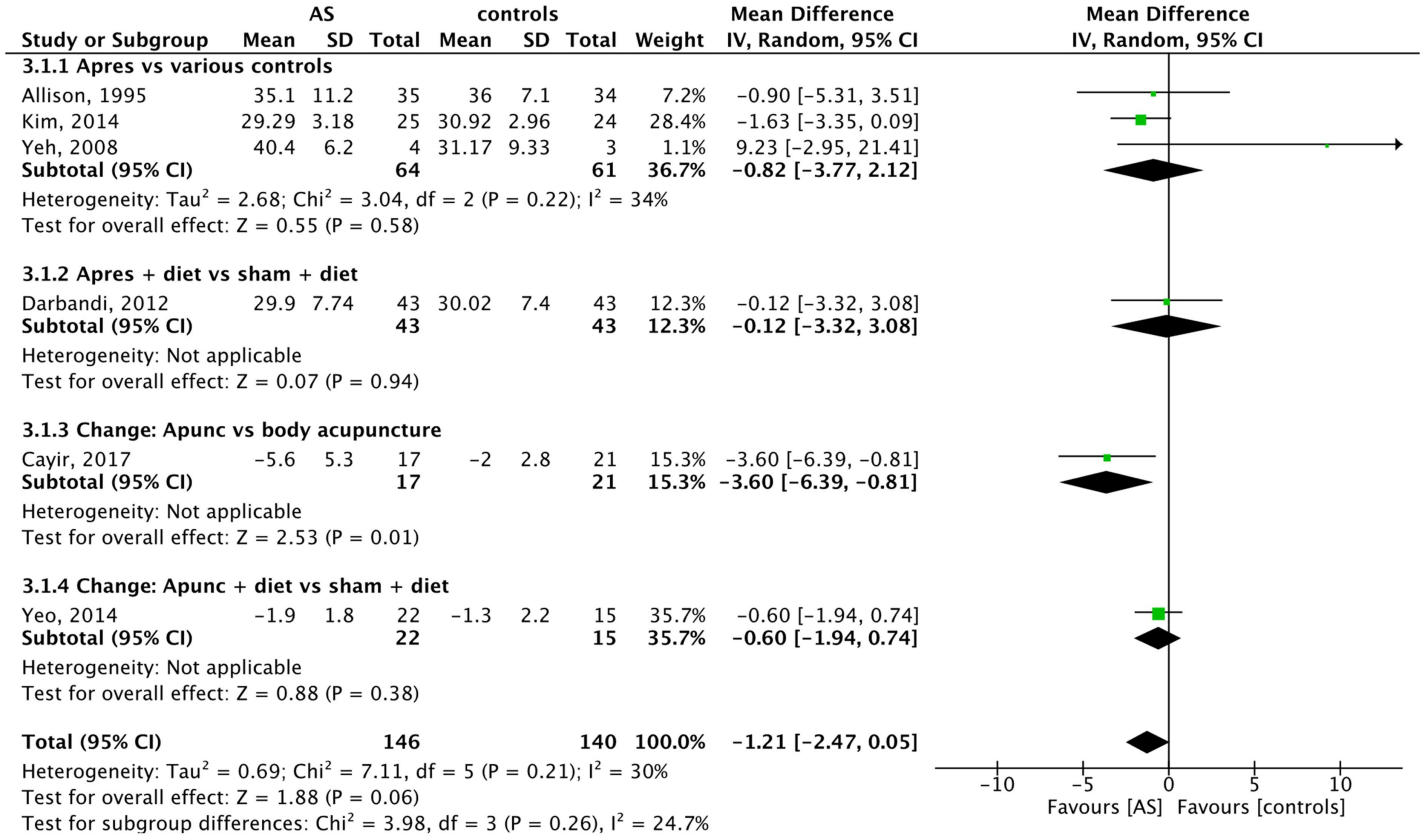
Body fat: AS vs. controls.

Data of 6 trials with 317 individuals were included in the analysis of body fat. In body fat percentage, compared to the control methods, AS did not show a significant effect on reducing the amount of body fat (MD = −1.21, 95% CI (−2.47 to 0.05), *p* = 0.06) (see Figure 5). Body fat was mostly measured with the method of impedance analysis. Cayir *et al* yielded the highest body fat percentage reduction from a baseline of 5.6% (SD5.3) with auricular acupuncture vs. 2.0% (SD2.8) with body acupuncture. No additional measures were used by the patients, and both acupuncture forms were applied two times per week over 12 weeks.

### 3.11 Waist circumference

**Figure 6:**
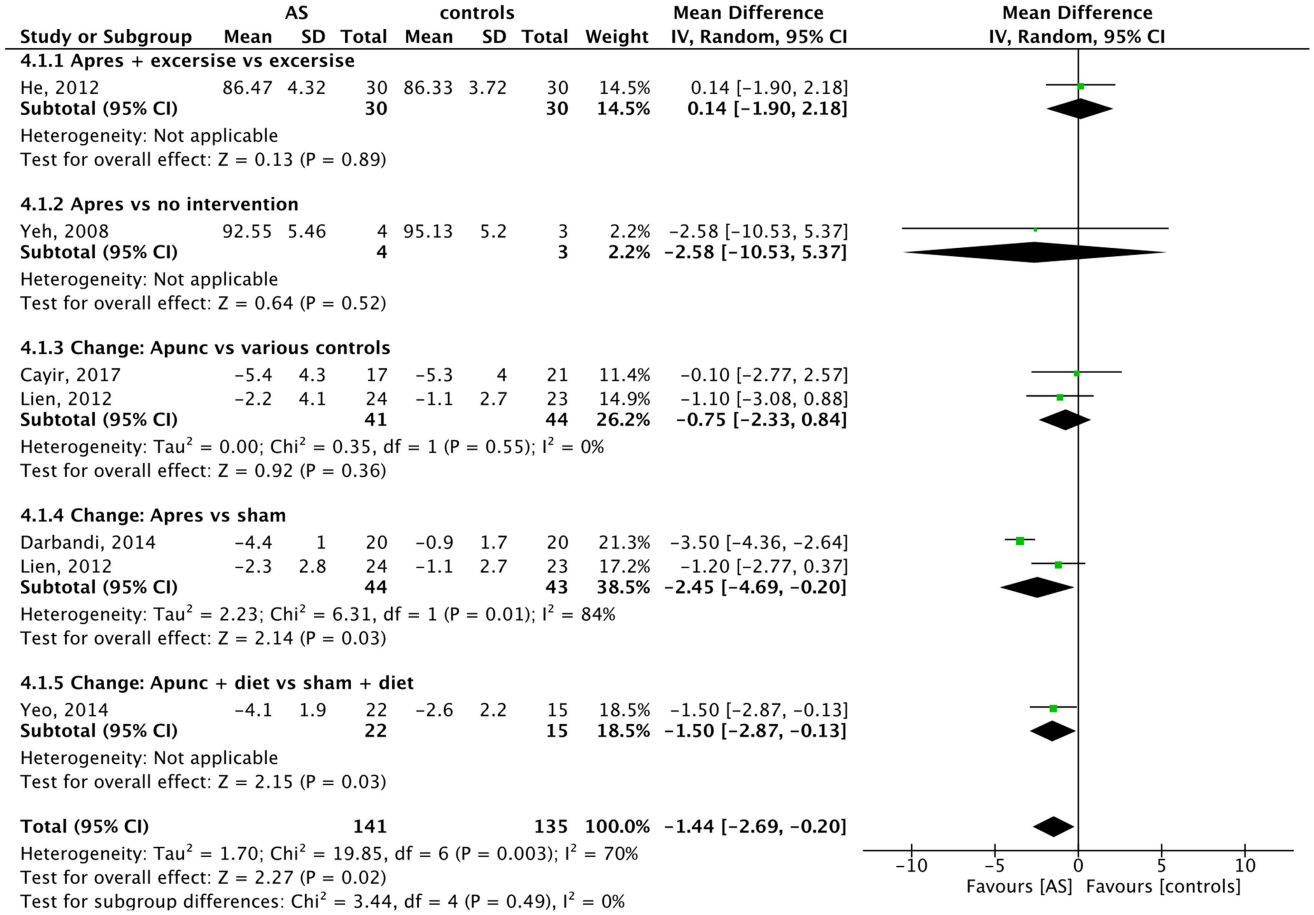
Waist circumference: AS vs. controls.

In the analysis of the six trials with data on waist circumference of 276 individuals, compared to the control methods, AS reduced waist circumference significantly (MD = −1.44 cm, 95% CI (−2.69 to −0.20), *p* = 0.02) (see Figure 6). The biggest decrease was achieved in the trial by Darbandi et al. (2014), who randomized 80 obese participants in four groups: body electroacupuncture (A), auricular acupressure (C), sham body electroacupuncture (B), and sham auricular acupressure (D). From the auricular acupressure vs. sham auricular acupressure arms (n = 2×20), it was possible to use the data for meta-analysis. All subjects received a 500 kcal low-calorie diet. Only the verum auricular acupressure led to a relevant reduction of hip and waist circumference after 6 weeks of treatment compared to sham (Darbandi et al., 2014).

Regarding the pooled data from four or fewer trials (see forest plots in the Supplementary material), a positive effect on obesity-related metabolic parameters was observed only in the comparisons of leptin, insulin, and HOMA insulin resistance. Compared to the control methods, AS had a significant effect on reducing leptin (4 trials, *n* = 295, SMD = −0.40, 95% CI (−0.63 to −0.17), *p* = 0.0008). Regarding insulin, AS had a significant effect on reducing fasting insulin serum level (3 trials, *n* = 139, MD = −3.50 UI/ml, 95% CI (−6.59 to −0.41), *p* = 0.03) and HOMA insulin resistance (3 trials, *n* = 139, MD = −1.06, 95% CI (−2.03 to −0.09), p = 0.03).

No significant effects of AS compared to control methods were found in the meta-analyses of hip circumference, ratio of waist/hip circumference, cholesterol, LDL, triglycerides, adiponectin, and ghrelin (see Supplementary material). Only one trial examined the impact of glucose levels in obese patients (Costa-Cavalcanti et al., 2018). No significant difference was reported between the groups.

### 3.12 Sensitivity analysis

For details, see Tables 3, 4. We performed two sensitivity analyses: First, positive outliers were excluded from the meta-analysis.

**Table 3:** Sensitivity analysis: heterogeneity.

Outcome	Deletion	Heterogeneity test		Meta-analysis results	
		*I*^2^-test	*P*-value	MD/SMD (95%CI)	*P*-value
Waist Circumference: AS vs. control	He et al. (2012)	0%	0.8	MD -1.0 [−1.79, −0.21]	0.01
Hip Circumference: AS vs. control	Hsu et al. (2009) and He et al. (2012)	0%	0.88	MD 0.49 [−0.90, 1.89]	0.49
HDL: AS vs. control	Qunli and Zhicheng (2005)	44%	0.17	MD 0.05 [−0.40, 0.50]	0.83
Leptin: AS vs. control	Shen et al. (2009)	12%	0.34	SMD -0.27 [−0.52, −0.02]	0.04

**Table 4:** Sensitivity analysis: additional therapy.

Outcome	Deletion	Heterogeneity test		Meta-analysis results	
		*I*^2^-test	*p*-value	MD (95%CI)	*P*-value
Body fat: AS vs. control	Yeh and Yeh (2008) and Hsieh (2010)	39%	0.18	−1.80 [−3.98, 0.38]	0.11
BMI: AS vs. control	Yeh and Yeh (2008), Hsieh (2010), and Cha and Park (2020)	0%	0.73	−0.29 [−0.43, −0.15]	0.0001
Weight: AS vs. control	Yeh and Yeh (2008), Hsieh (2010), and Cha and Park (2020)	0%	0.77	−0.69 [−1.19, −0.19]	0.006
Waist Circumference: AS vs. control	Hsieh (2010) and Cha and Park (2020)	0%	0.88	−1.01 [−2.11, 0.10]	0.07

Second, trials with additional interventions besides AS were excluded. In the case of waist circumference and leptin, significant results could be still obtained, excluding positive outliers.

Regarding the sensitivity analysis of AS, excluding additional interventions such as dietary advice or physical exercise, we still found a significant benefit of AS alone for reducing BMI and weight.

### 3.13 Descriptive analysis of studies

There were seven trials that did not have continuous data for quantitative meta-analysis. In Table 5, the dichotomous outcomes are summarized.

**Table 5:** Results of descriptive analysis.

Outcome and No of RCTs	Only in AS group
Body weight N = 5	Significant reduction: 3 [19, 24, 26] Non-significant reduction: 2 [29, 32]
BMI N = 5	Significant reduction: 3 [13, 14, 19] Non-significant reduction: 2 [18, 32]
Waist circumference N = 1	Significant reduction: 1 [32]
Body fat N = 1	Significant reduction: 1 [19]
Blood glucose N = 1	Non-significant reduction: 1 [33]

### 3.14 Safety of intervention

Of the 22 included studies, adverse events were recorded in 12 studies. Six study groups could not document any adverse side effects (Mok et al., 1976; Yeh and Yeh, 2008; Darbandi et al., 2012, 2014; Cayir et al., 2017; Cha and Park, 2020). Minor side effects were recorded in five trials (Allison et al., 1995; Hsu et al., 2009; Ching et al., 2012; Lien et al., 2012; Schukro et al., 2014). One study described in its methodology that they recorded adverse side effects. However, no information could be extracted from the results (Yeh et al., 2015).

Common side effects of AS that have been reported are all mild and include pain or minor bleeding at the stimulation side (four trials), transient dizziness (three trials), erythema (two trials), and skin irritation (two trials).

### 3.15 Quality assessment

**Figure 7:**
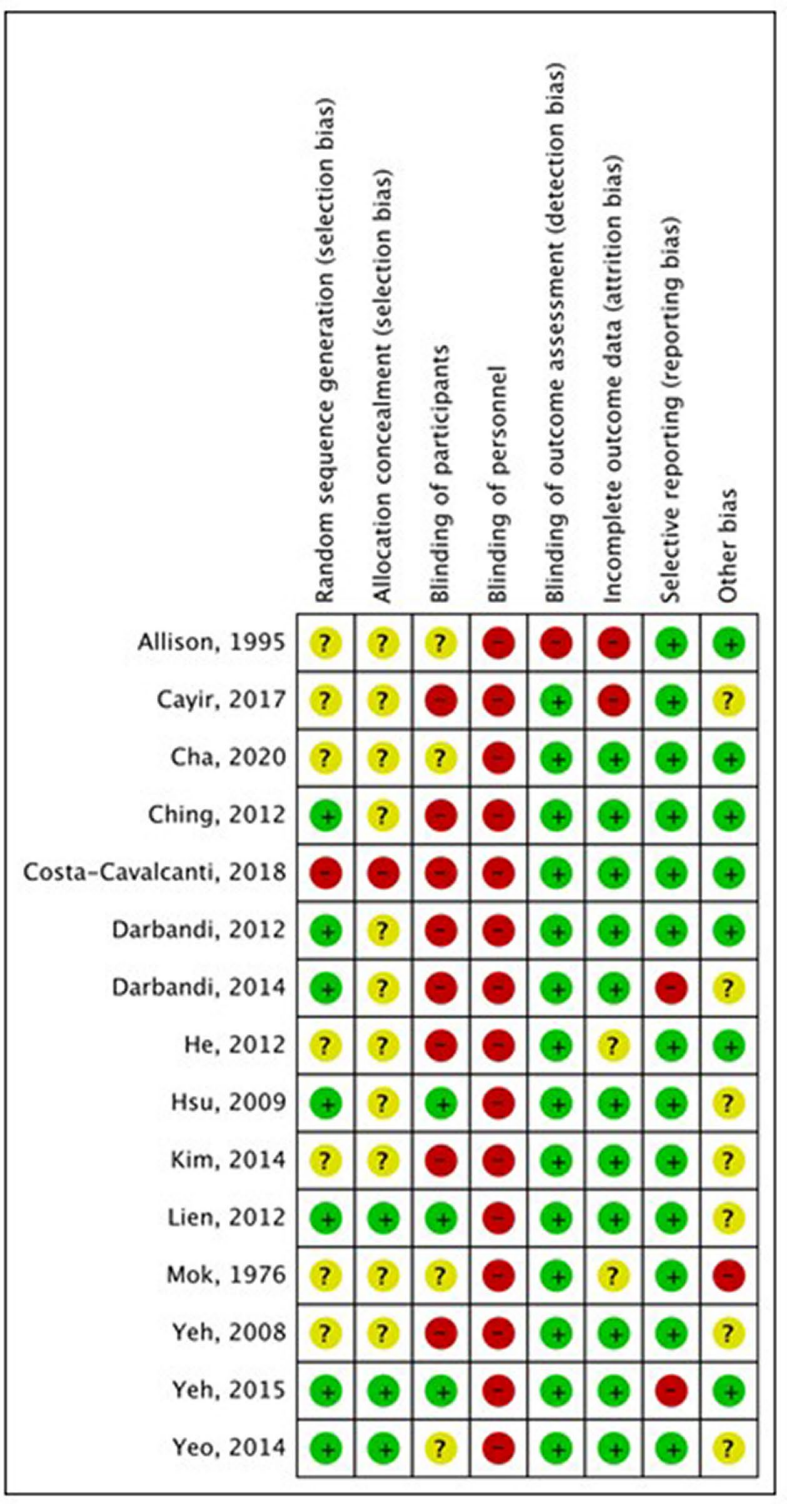
Risk of bias assessment. +: low risk of bias; –: high risk of bias;?: unclear risk of bias.

**Figure 8:**
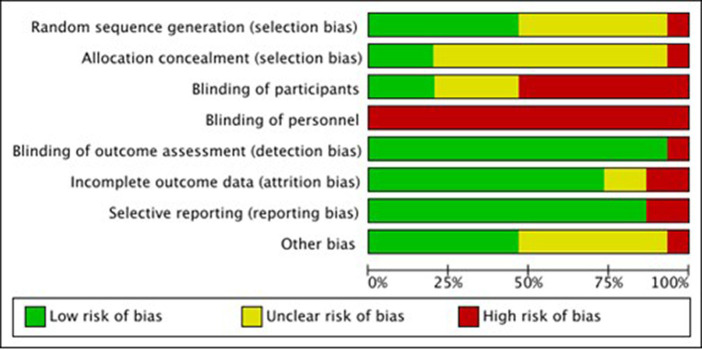
Risk of bias summary.

The overall study quality was moderate (see Figures 7, 8). A funnel plot was performed and revealed no publication bias.

### 3.16 Excluded studies

One trial had several contradictions in the tables, and we judged it prudent to exclude it from the evaluation (Abdi et al., 2012).

### 3.17 Funding sources

Most study groups had not provided funding information (Mok et al., 1976; Qunli and Zhicheng, 2005; Hsieh, 2007, 2010; Shen et al., 2009; Hsieh et al., 2011; Lien et al., 2012; Kim et al., 2014; Schukro et al., 2014). For the most part, the studies were supported either by the research group itself (Hsu et al., 2009; Darbandi et al., 2012, 2014; Yeh et al., 2015; Cayir et al., 2017; Lillingston et al., 2019) or by the government (Yeh and Yeh, 2008; Ching et al., 2012; Yeo et al., 2014; Costa-Cavalcanti et al., 2018; Cha and Park, 2020). Individual studies were funded by a company (Allison et al., 1995) or an acupuncture society (He et al., 2012).

## 4 Discussion

The results of the meta-analysis demonstrated that a reduction in weight and BMI compared to controls was achieved by AS of the auricular regions that receive afferent vagal nerve supply in obese patients. The achieved mean reductions measured—despite additional diets, exercise programs, and a mean of 50 days of treatment—were marginal, and did not reach the 5% reduction of body weight that is considered clinically meaningful (Williamson et al., 2015).

The weight reduction achieved in this meta-analysis is in the range of physiological weight fluctuations over the day (Simkin-Silverman et al., 1998; Field et al., 2001); on the other hand, some trials offered exercise programs for both the intervention and control groups, so an increase in muscle mass might have concealed the loss of body fat in the weight measurements. Regarding obesity-related hormones, the clinical significance of the results remains elusive due to the small sample size.

Interestingly, acupressure had comparable or bigger effects than acupuncture, possibly due to the continuous stimulation, as the pellets remained plastered *in situ* for weeks and patients were instructed to press them daily. This effect has been observed in past research on AS (Usichenko et al., 2022). This effect, though, could have had an impact on sham needles with a missing needle tip. The plasters contain a metal ring, which might have had a stimulating effect, too, when massaged.

In the analysis of the safety aspects of the different forms of AS, only minor side effects have been reported in the included studies, which is in line with the results of a review of reviews of the risks and safety of extended auricular therapy (Nielsen et al., 2020). Throughout the trials, a moderate quality was found. The analysis of study quality was performed with the RoB tool 1 from the Cochrane handbook, which was chosen for reasons of stricter judgment.

Interestingly, a relevant weight reduction was not achieved by diets either; this fact has been described before in other systematic reviews comparing different diets (Naude et al., 2022).

Exercise seems more promising–a recently published systematic review with meta-analysis investigated the influence of dancing compared to no intervention on the parameters of obesity in 10 RCTs with 650 overweight and obese participants. The dancing corresponded to the weekly amount of exercise recommended by the World Health Organization (WHO) (150–300 min). The duration of the intervention was 42–84 days. They were able to achieve an average BMI reduction of 1.03 kg/m2 (95% CI -1.63 to −0.44; *p* = 0.0006) (Zhang et al., 2024).

The effects of vagal stimulation at the auricle on stress response and cardiovascular parameters have been proven in numerous previous studies (Qu et al., 2014; Usichenko et al., 2022; Hua et al., 2023; Sigrist et al., 2023). It remains to be determined, if the mechanism behind the weight loss via AS is linked to an altered stress response, in the face of calorie reduction.

The strength of this review is that it included more RCTs and more anthropometric and blood chemistry values than previous reviews and it presents an overview of the most frequently stimulated points. The present findings are comparable to a systematic review and meta-analysis published in 2020, in which the effects of AS were analyzed in a smaller sample of trials with limited outcomes (Mendonça et al., 2020). In the meta-analysis of five trials, BMI was reduced by a mean of 0.86 kg/m2 (95% CI, 0.533–1.196; *p* < 0.004) and weight by 1.5 kg (95% CI, 0.606–2.407; *p* < 0.0001). In this review by Mendoca et al., the mean achieved body weight reduction still does not reach a clinically significant 5% of body weight, nor does it reach a full point of BMI in the calculated total difference (Mendonça et al., 2020). Limitations: A limiting factor to the present systematic review is that only studies in English were finally included in this analysis. Auriculotherapy has long been used in traditional medical systems such as Chinese medicine; however, scientific contributions in Chinese, Korean, and Japanese are missing in this review, and their influence on the evidence remains unclear. Although we tried to compare the outcomes that were assessed at the end of the treatment, there is substantial heterogeneity in the duration of intervention, which might be a further limitation. Regarding the heterogeneity of control groups, we tried to account for it with clustering in subgroups. Another limitation of the results of this review is the inclusion of sham controls that might not be inert. Though the trial might give an indicator of the specific effects of certain regions of the auricle, every stimulation of the auricle, no matter the region, leads to sensory afferences leading to some kind of body reaction. This issue has been discussed extensively in the literature regarding sham points in body acupuncture (Lee et al., 2023, 2024).

Future ear acupuncture trials should take into account that there are no “inert” regions on the auricle, and even an “empty” plaster as sham control might exert an effect when massaged. Therefore, while the effect does not appear to be clinically relevant, the size of the effect may be underestimated due to active sham trials. Another bias is the high possibility of unblinding as a result of the long duration of stimulation (Usichenko and Cummings, 2024), such as plasters falling off to reveal the status of sham or real needles.

## 5 Conclusion

This study suggests an effect of AS in the treatment of obesity, but the size of the effect does not appear to be of clinical relevance, though it may be underestimated due to active sham trials. Regarding the influence of AS on metabolic parameters such as blood lipids and obesity-related hormones, more research is needed.

## Data availability statement

The original contributions presented in the study are included in the article, further inquiries can be directed to the corresponding author/s.

## Author contributions

KH: Data curation, Formal analysis, Investigation, Writing – original draft. TU: Conceptualization, Supervision, Validation, Writing – review & editing. MC: Conceptualization, Methodology, Validation, Writing – review & editing. MB: Writing – review & editing. SW: Methodology, Supervision, Validation, Writing – review & editing. BB: Funding acquisition, Supervision, Validation, Writing – review & editing. JD: Conceptualization, Formal analysis, Investigation, Project administration, Resources, Supervision, Writing – original draft.
