# Supplementary material for: Effects of auricular stimulation on weight- and obesity-related parameters: a systematic review and meta-analysis of randomized controlled clinical trials
Source: Front Neurosci. 2024 Aug 6;18:1393826. doi: 10.3389/fnins.2024.1393826 (PMC11333859; doi:10.3389/fnins.2024.1393826)
Supplement: Supplementary file 1 [file Data_Sheet_1.zip › Supplement 1.DOCX]

Forest plots will be presented for pooled outcome parameters of 5 trials and more in the main publication, forest plots of 4 and less trials in the supplementary material.


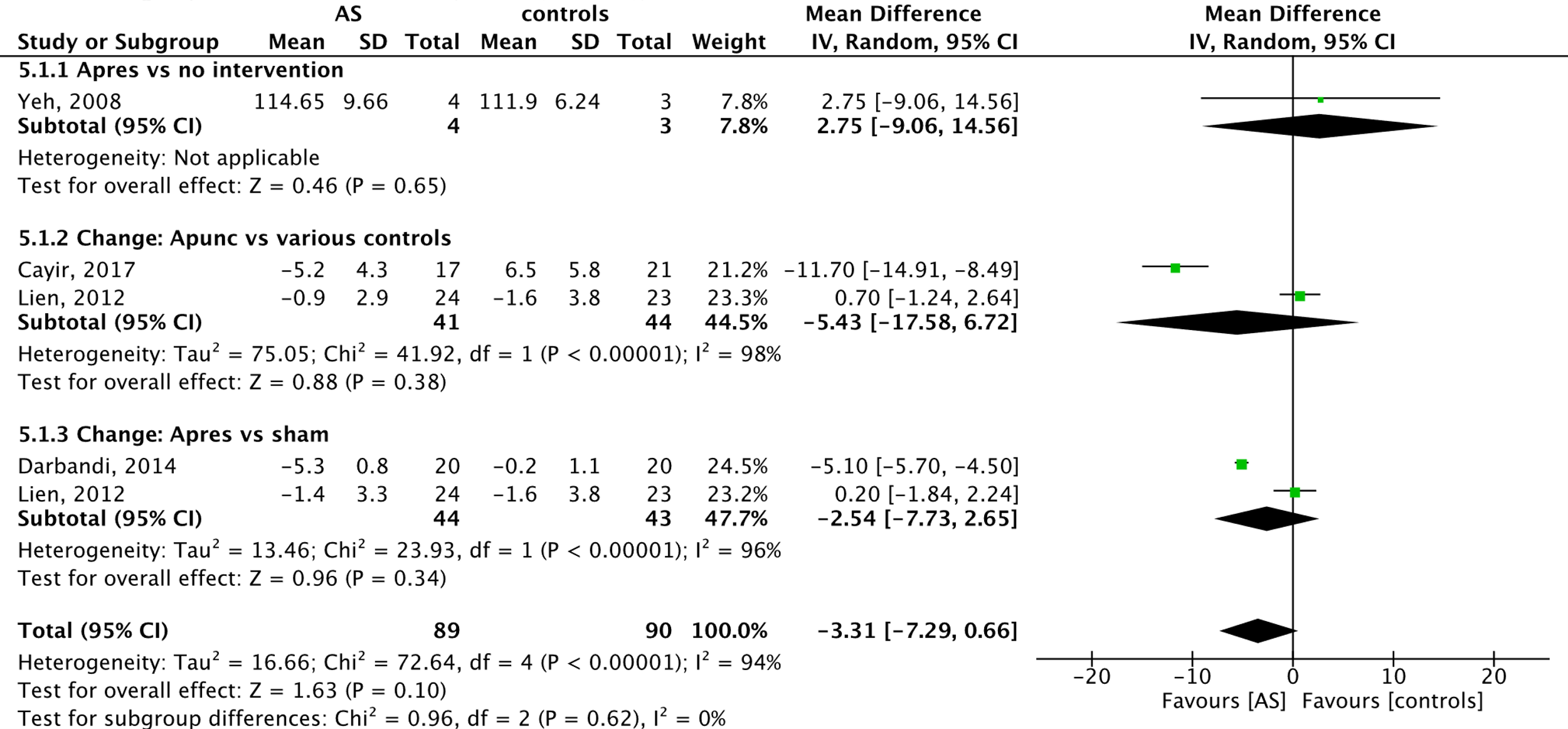


Supp 1 - Hip Circumference: Auricular Stimulation vs. controls
