# Supplementary material for: Effects of auricular stimulation on weight- and obesity-related parameters: a systematic review and meta-analysis of randomized controlled clinical trials
Source: Front Neurosci. 2024 Aug 6;18:1393826. doi: 10.3389/fnins.2024.1393826 (PMC11333859; doi:10.3389/fnins.2024.1393826)
Supplement: Supplementary file 1 [file Data_Sheet_1.zip › Supplement 9, 10, 11.DOCX]

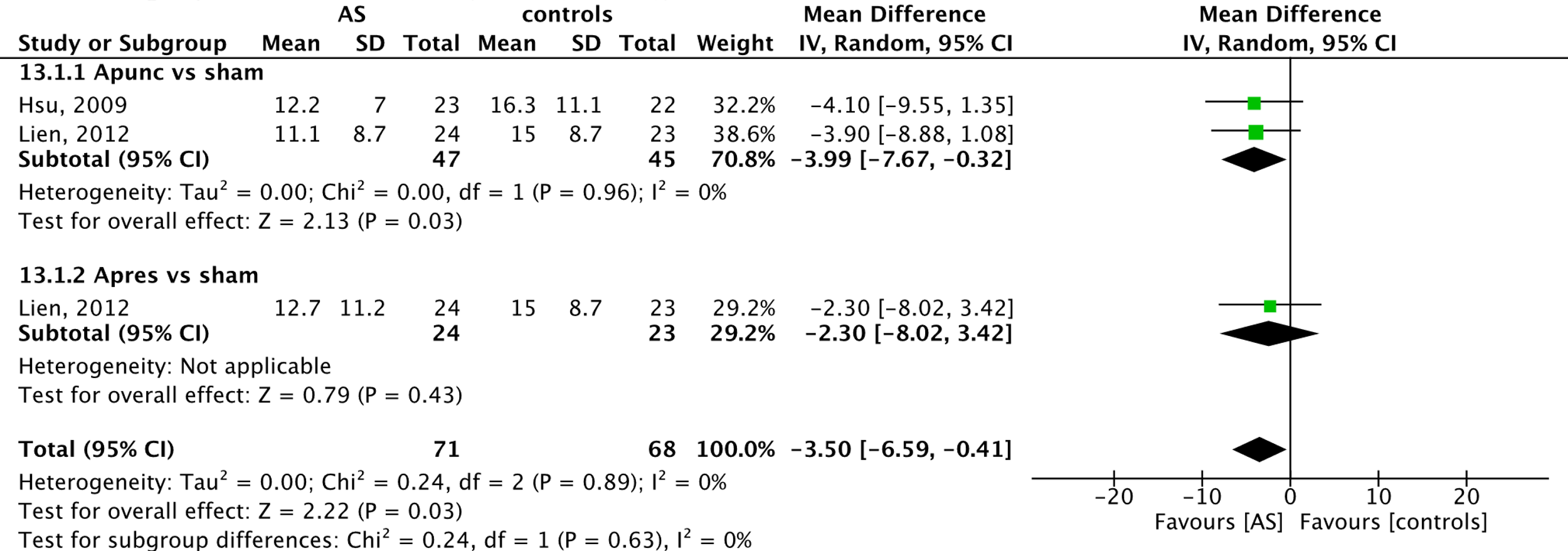


Supp 9 - Insulin: Auricular Stimulation vs. controls


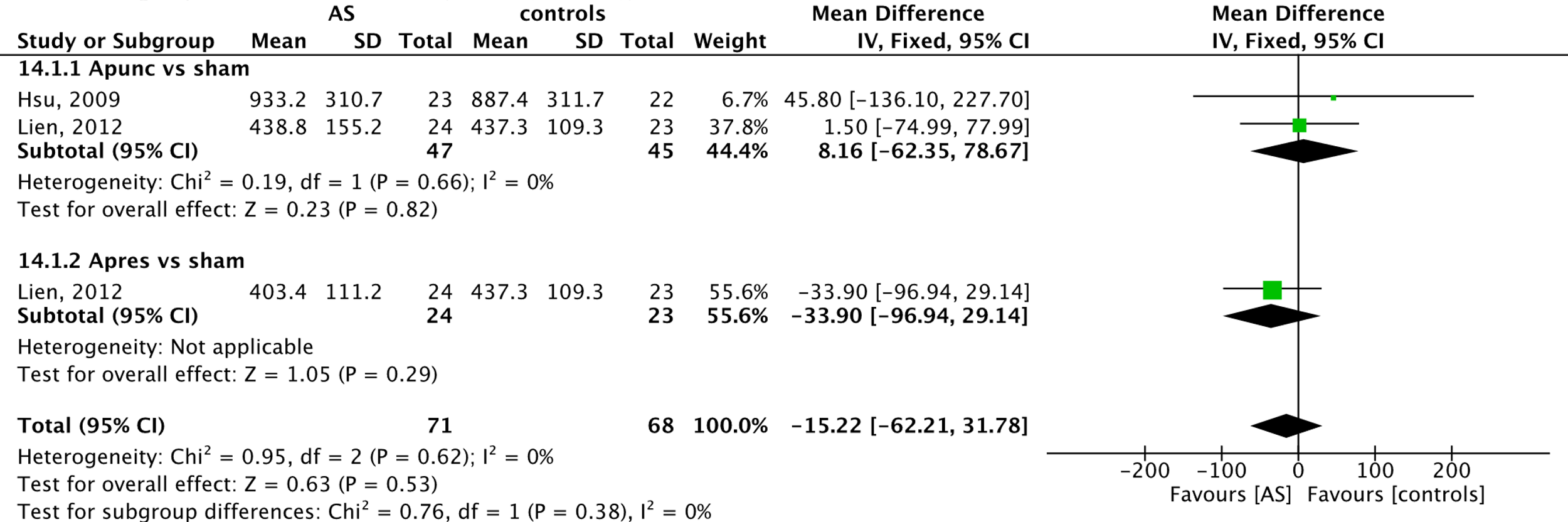


Supp 10 - Ghrelin: Auricular Stimulation vs. controls


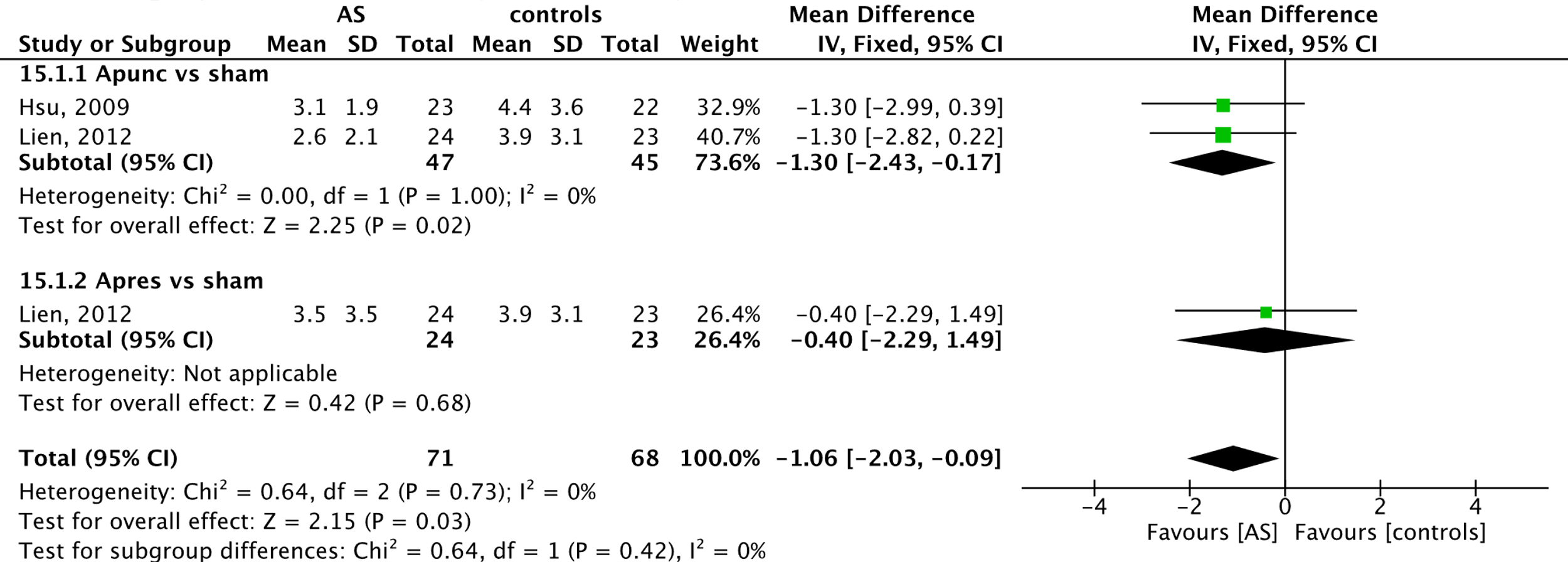


Supp 11 – HOMA insulin resistance: Auricular Stimulation vs. controls
