# Supplementary material for: Effects of auricular stimulation on weight- and obesity-related parameters: a systematic review and meta-analysis of randomized controlled clinical trials
Source: Front Neurosci. 2024 Aug 6;18:1393826. doi: 10.3389/fnins.2024.1393826 (PMC11333859; doi:10.3389/fnins.2024.1393826)
Supplement: Supplementary file 1 [file Data_Sheet_1.zip › Supplement 12.DOCX]

Supp 12 – Results GRADE Evaluation

**CI:** confidence interval; **MD:** mean difference**; SMD:** standardised mean difference

#### Explanations

1. Two studies show a high risk of bias. They have a high risk of bias in three criteria. In addition, five studies were included that recorded a high risk of bias in two qualities. (maximum high risk of bias on "blinding personal")
2. The higher confidence intervall breaches our MD 0.2 taken as clinically important differences MCID vs control
3. Two studies show a high risk of bias. They have a high risk of bias in three criteria. In addition, six studies were included that recorded a high risk of bias in two qualities. (maximum high risk of bias on "blinding personal")
4. Two studies show a high risk of bias. They have a high risk of bias in three criteria. In addition, three studies were included that recorded a high risk of bias in two qualities. (maximum high risk of bias on "blinding personal")
5. The higher confidence intervall breaches our SMD 0.2 taken as clinically important differences MCID vs control and also breaches the line of no effect.
6. Two studies show a high risk of bias. They have a high risk of bias in three criteria. In addition, two studies were included that recorded a high risk of bias in two qualities. (maximum high risk of bias on "blinding personal")
7. High heterogenity is detected (I^2^ = 70%).
8. Two studies show a high risk of bias. They have a high risk of bias in three criteria. In addition, one studiy was included that recorded a high risk of bias in two qualities. (maximum high risk of bias on "blinding personal")
9. High heterogenity is detected (I^2^ = 94%).
10. Two studies show a high risk of bias. They have a high risk of bias in two criteria. (maximum high risk of bias on "blinding personal")
11. One study shows a high risk of bias. He has a high risk of bias in four criteria. In addition, one study was included that recorded a high risk of bias in two qualities. (maximum high risk of bias on "blinding personal")
12. One study shows a high risk of bias. He has a high risk of bias in four criteria. (maximum high risk of bias on "blinding personal")
13. One study was included that recorded a high risk of bias in two qualities. (maximum high risk of bias on "blinding personal")
14. High heterogenity is detected (I^2^ = 82%).
15. High heterogenity is detected (I^2^ = 52%).
16. High heterogenity is detected (I^2^ = 64%).
