# Supplementary figures and images for: Effects of auricular stimulation on weight- and obesity-related parameters: a systematic review and meta-analysis of randomized controlled clinical trials
Source: Front Neurosci. 2024 Aug 6;18:1393826. doi: 10.3389/fnins.2024.1393826 (PMC11333859; doi:10.3389/fnins.2024.1393826)

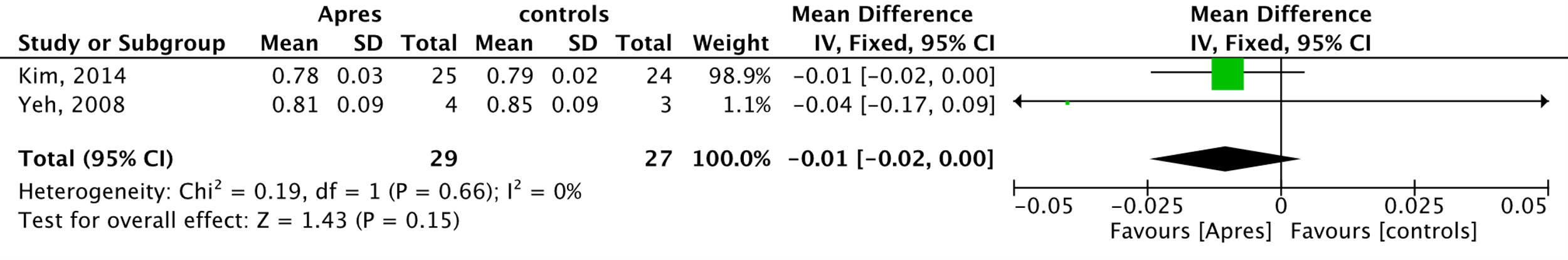


Supp 2 - Waist/Hip Circumference Ratio: Auricular Acupressure vs. controls

Supplement: Supplementary file 1 [file Data_Sheet_1.zip › Supplement 2.DOCX]

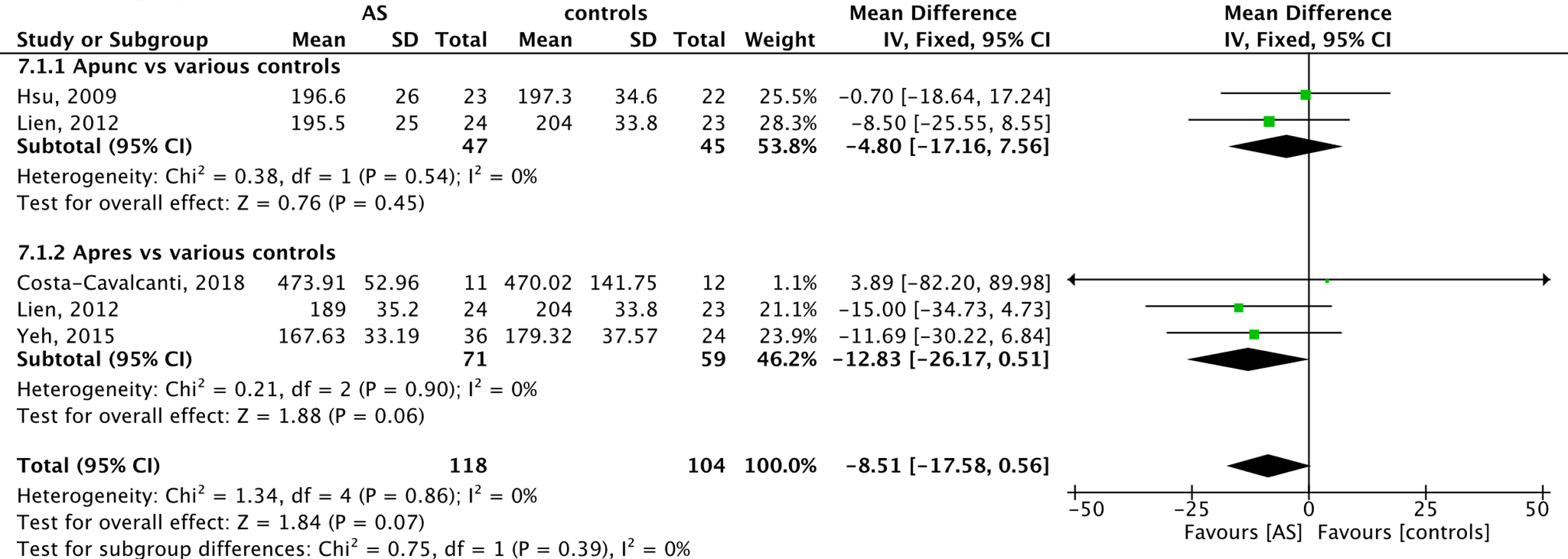


Supp 3 - Cholesterol: Auricular Stimulation vs. controls

Supplement: Supplementary file 1 [file Data_Sheet_1.zip › Supplement 3.DOCX]

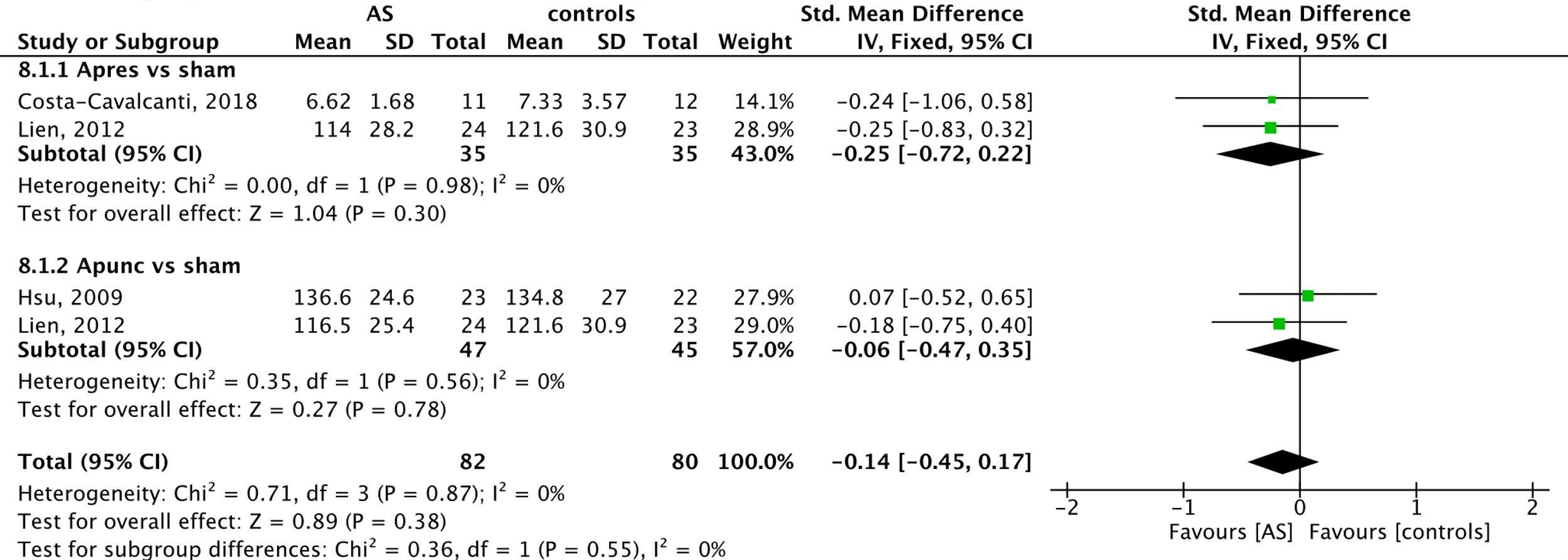


Supp 4 - LDL: Auricular Stimulation vs. controls

Supplement: Supplementary file 1 [file Data_Sheet_1.zip › Supplement 4.DOCX]

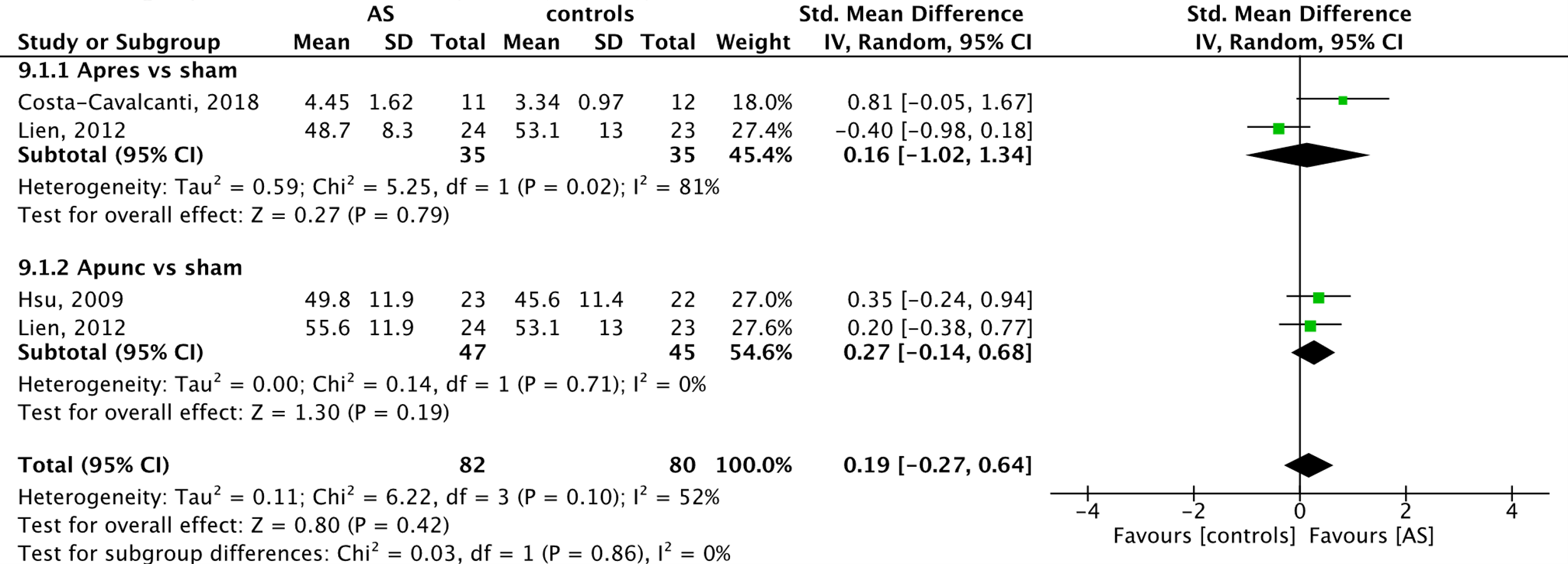


Supp 5 - HDL: Auricular Stimulation vs. controls

Supplement: Supplementary file 1 [file Data_Sheet_1.zip › Supplement 5.DOCX]

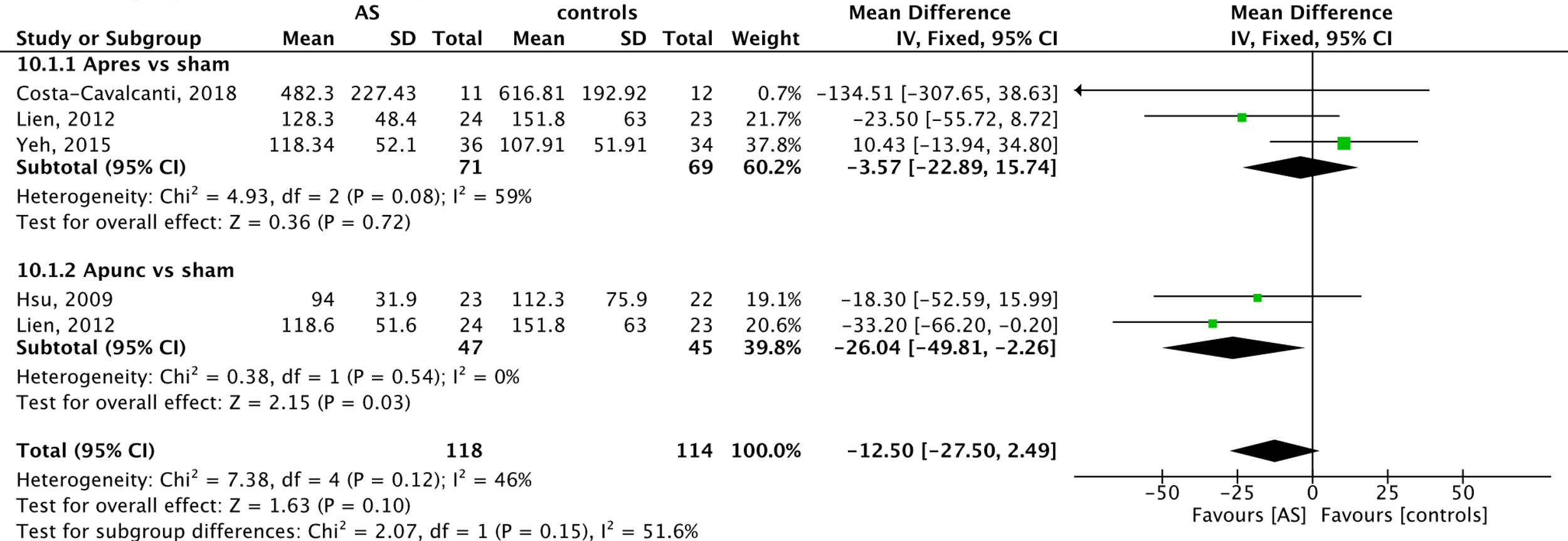


Supp 6 - Triglyceride: Auricular Stimulation vs. controls

Supplement: Supplementary file 1 [file Data_Sheet_1.zip › Supplement 6.DOCX]

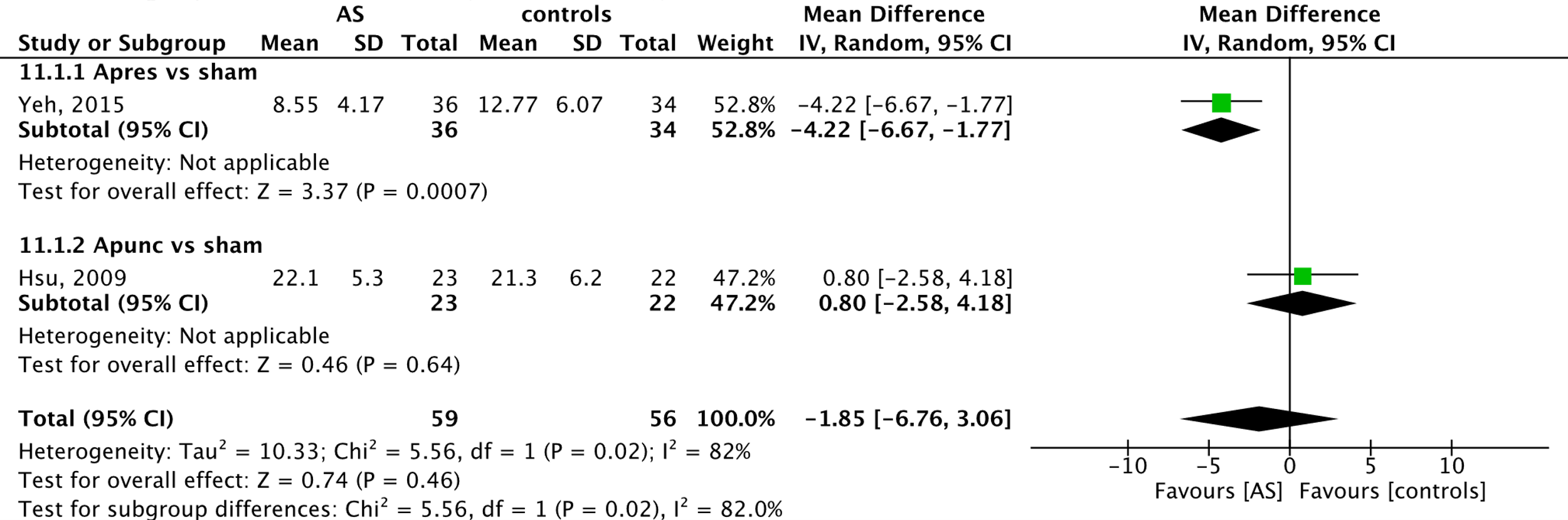


Supp 7 - Adiponectin: Auricular Stimulation vs. controls

Supplement: Supplementary file 1 [file Data_Sheet_1.zip › Supplement 7.DOCX]

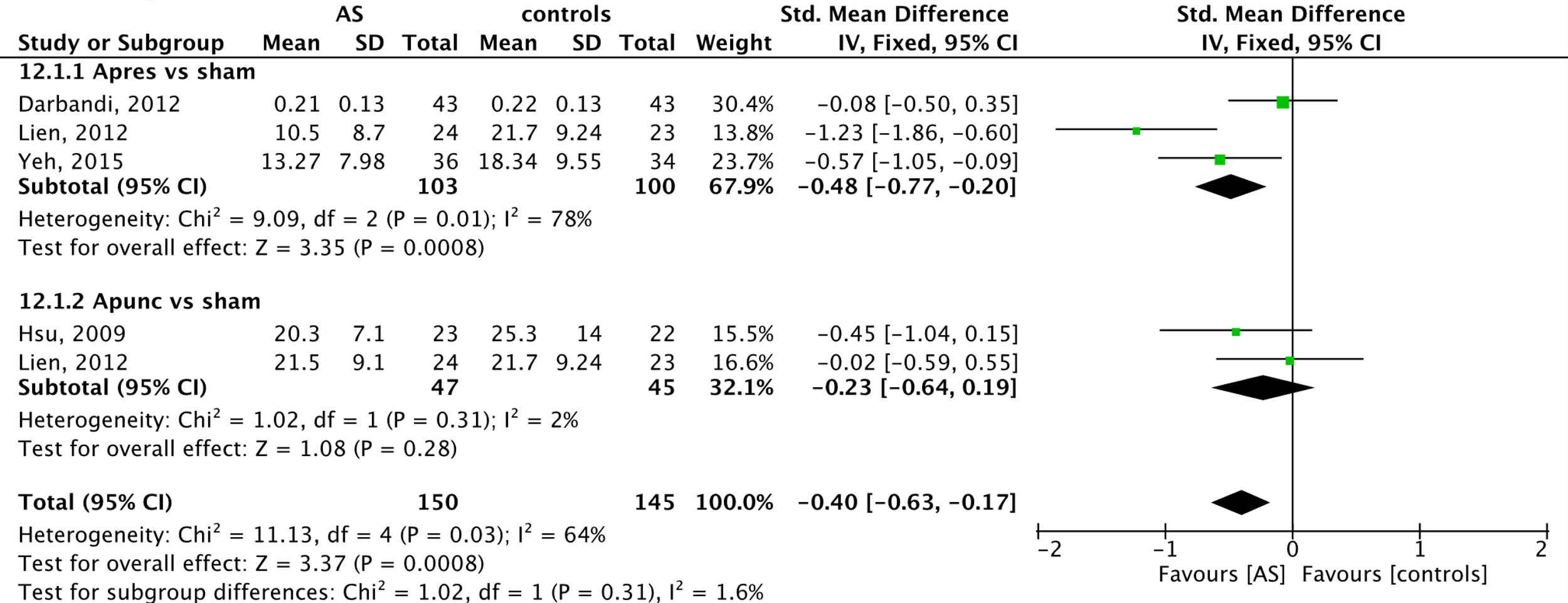


Supp 8 - Leptin: Auricular Stimulation vs. controls

Supplement: Supplementary file 1 [file Data_Sheet_1.zip › Supplement 8.DOCX]
